# Systemic inflammation, microbiology, and clinical outcomes in eosinophilic and non-eosinophilic acute exacerbations of chronic obstructive pulmonary disease

**DOI:** 10.3389/fmed.2026.1917271

**Published:** 2026-08-27

**Authors:** Ning Zhang, Qiqiang Zhou, Sujie Zhao, Chen Zhang, Yahong Chen, Ying Liang, Yongchang Sun

**Affiliations:** 1 Department of Respiratory and Critical Care Medicine, Peking University Third Hospital, Beijing, China; 2 Research Center for Chronic Airway Diseases, Peking University Health Science Center, Beijing, China; 3 Department of Respiratory and Critical Care Medicine, Lulong County Hospital, Qinhuangdao, Hebei, China; 4 Information Management and Big Data Center, Peking University Third Hospital, Beijing, China

**Keywords:** acute exacerbation, chronic obstructive pulmonary disease, eosinophils, prognosis, systemic inflammation

## Abstract

**Objective:**

Blood eosinophil (EOS) guides corticosteroid therapy in stable chronic obstructive pulmonary disease (COPD), but its prognostic value in COPD exacerbation (ECOPD) remains controversial. This study characterized clinical features and assessed the impact of EOS levels on clinical outcomes in hospitalized ECOPD patients.

**Methods:**

We retrospectively reviewed hospitalized patients with ECOPD between 2018 and 2023. Patients were categorized according to percentage of peripheral blood eosinophil: the eosinophilic group (EOS ≥ 2%) and the non-EOS group (EOS < 2%). The primary outcome was in-hospital mortality, while the secondary outcomes included ICU admission, mechanical ventilation, and length of hospital stay.

**Results:**

Among 511 patients (EOS group, *n* = 139; non-EOS group, *n* = 372), the EOS group had lower levels of inflammatory markers (C-reactive protein and procalcitonin) and a lower detection rate of Gram-negative bacilli, especially *Acinetobacter baumannii*. The EOS group exhibited significantly lower rates of ICU admission (25.18% vs. 50.27%; *P* < 0.001) and in-hospital mortality (1.44% vs. 6.72%; *p* = 0.018). In the multivariable analyses, EOS% ≥ 2% remained associated with lower odds of in-hospital mortality after adjustment for age and heart failure (adjusted OR, 0.225; 95% CI, 0.052–0.971; *p* = 0.046) and with lower odds of ICU admission after adjustment for age, sex, and heart failure (adjusted OR, 0.357; 95% CI, 0.229–0.556; *p* < 0.001).

**Conclusion:**

Among hospitalized patients with ECOPD, elevated peripheral blood eosinophils were associated with favorable clinical outcomes, especially a reduced risk of ICU admission, as well as a lower systemic inflammation burden.

## 1 Introduction

Chronic obstructive pulmonary disease (COPD) is a prevalent chronic airway disease with high mortality, posing a significant threat to public health. Exacerbations of COPD (ECOPD) represent critical events in the progression of the disease. Frequent exacerbations not only accelerate the decline in lung function, but also significantly increase hospitalization rates and mortality (1). During acute exacerbations, patients face a higher risk of complications, such as respiratory failure and cor pulmonale, which pose a serious threat to their life. Therefore, identifying biomarkers that reflect disease activity, guide treatment decisions, and predict prognosis is crucial for clinical risk assessment, personalized treatment, and improving long-term management.

Eosinophil (EOS) is a biomarker of type 2 inflammation in the airways and can help guide the use of inhaled corticosteroids in patients with stable COPD. A higher blood EOS count predicts greater lung function decline and the risk of future exacerbations. Data from the COPDGene and ECLIPSE studies indicate that the risk of exacerbation increases with higher peripheral blood EOS counts and percentages. Specifically, the risk of acute exacerbation rises by 22% when blood EOS is ≥2% (2). Additionally, patients with blood EOS ≥ 2% or 200 cells/μL have an increased frequency of readmissions or emergency department visits due to ECOPD within 1 year, and they experience shorter readmission times (3). However, some prospective studies have shown no significant difference in the proportion of patients experiencing moderate-to-severe exacerbations over a follow-up period of ≥5 years, whether their blood EOS remained consistently ≥150 cells/μL, consistently ≤150 cells/μL, or fluctuated around 150 cells/μL (4). A meta-analysis encompassing 21 studies with 18,041 patients found that COPD patients with increased EOS during acute exacerbations had lower mortality and shorter hospital stays but a higher risk of readmission (5). These conflicting results suggest that the role of EOS on predicting disease severity and prognosis during ECOPD still requires further clarification. It is important to note that ECOPD are often triggered by respiratory tract infections, and phenotype of airway inflammation may be closely related to the lower respiratory tract microbiome characteristics (6). Elevated EOS levels may indicate a lower bacterial infection burden and a relatively “non-bacterial” phenotype of acute exacerbation (7), although the evidence remains insufficient. Additionally, there is a lack of large-scale studies to support whether the spectrum of lower respiratory tract pathogens in hospitalized ECOPD patients varies with EOS levels.

Given the current controversies regarding the relationship between blood EOS levels and clinical outcomes in hospitalized patients with ECOPD, this study aimed to conduct a retrospective analysis of a larger cohort of hospitalized ECOPD patients, and to compare the clinical characteristics, microbiological features, and the impact on in-hospital outcomes between patients with eosinophilic and non-eosinophilic phenotypes.

## 2 Materials and methods

### 2.1 Study subjects and data collection

This study consecutively included patients hospitalized for ECOPD between January 2018 and December 2023 at the Department of Respiratory and Critical Care Medicine, Peking University Third Hospital. All patients met the Global Initiative for Chronic Obstructive Lung Disease (GOLD) criteria (8) for diagnosis of ECOPD characterized by worsening respiratory symptoms including dyspnea and/or cough and purulent sputum. Additional inclusion criteria were as follows: age 40 years or older, having complete clinical data, including detailed medical history and comprehensive laboratory test results. If a patient had ≥2 hospitalizations during the study period, clinical data from the first hospitalization were collected. The exclusion criteria were as follows: (1) Coexisting other severe pulmonary diseases, such as bronchial asthma, cystic fibrosis, pulmonary embolism, or bronchiectasis. (2) Presence of malignancies, autoimmune diseases, or hematological disorders. (3) Use of systemic corticosteroids, immunosuppressants, or other drugs that may affect blood eosinophil levels within 48 h prior to hospitalization. (4) Severe liver or kidney dysfunction. (5) Presence of severe trauma or stress-related conditions. During the study period, patients diagnosed with COVID-19 or influenza were triaged through the fever clinic and were admitted to the Department of Infectious Diseases rather than our department from which the present cohort was derived. Therefore, patients with diagnosed COVID-19 or influenza were not included in the source population of this study. A complete-case approach was used. Patients with missing data for any of the clinical, laboratory or outcome variables required for the present analyses were excluded during cohort screening. All clinical and laboratory data were extracted from the Electronic Data Capture (EDC) system by the Information Center of Peking University Third Hospital.

After collection of the baseline blood sample, patients received treatment according to GOLD guideline recommendations (8). Nebulized bronchodilators, as well as systemic corticosteroids or nebulized inhaled corticosteroids, were administered, and antibiotics were prescribed when standard indications were met, including: (1) the simultaneous presence of all three cardinal symptoms: worsening dyspnea, increased sputum volume, and purulent sputum; (2) the presence of any two of these symptoms, with purulent sputum being one of them; or (3) the need for invasive or non-invasive mechanical ventilation. ICU admission was considered according to one of the following criteria: severe dyspnea unresponsive to initial treatment; altered mental status; persistent or worsening hypoxemia despite oxygen therapy and non-invasive ventilation (PaO₂ < 40 mmHg); severe or worsening respiratory acidosis (pH < 7.25); a requirement for invasive mechanical ventilation; or hemodynamic instability requiring vasoactive medication.

Clinical data of patients were retrospectively reviewed and collected through the electronic medical record system of Peking University Third Hospital. The data included patient demographics (age, gender), comorbidities, treatment details (whether ICU admission was required, use of invasive or non-invasive ventilation, medication prescribed, etc.), and length of hospital stay.

Laboratory indicators included values from routine blood tests (white blood cell count, red blood cell count, hemoglobin, hematocrit, platelet count, neutrophil count and percentage, eosinophil count and percentage, and lymphocyte count and percentage), as well as C-reactive protein (CRP), procalcitonin (PCT), D-dimer, and N-terminal pro B-type natriuretic peptide (NT-proBNP). Blood samples for all the aforementioned laboratory measurements were collected within 24 h of hospital admission and before the initiation of systemic corticosteroid treatment. Complete blood counts were measured using a Sysmex XN-series automated hematology analysis system (Sysmex Corporation, Kobe, Japan) using sheath-flow impedance and flow cytometric light-scattering methods. C-reactive protein (CRP) was measured using an IMMAGE 800 immunochemistry system (Beckman Coulter, Brea, CA, USA) with an immunoturbidimetric assay. Procalcitonin (PCT) and N-terminal pro-B-type natriuretic peptide (NT-proBNP) were measured using a Cobas e 601 electrochemiluminescence immunoassay system (Roche Diagnostics, Mannheim, Germany). D-dimer was measured using a STA-R-series automated coagulation analyzer (Diagnostica Stago, Asnières-sur-Seine, France) with a latex-enhanced immunoturbidimetric assay. All laboratory measurements were performed in accordance with the manufacturers’ instructions and the laboratory’s standard operating procedures. The clinical laboratory was accredited in accordance with ISO 15189.

Additionally, all patients included in the analysis underwent conventional bacterial sputum culture within 72 h after hospital admission. Sputum specimens were evaluated microscopically by the clinical microbiology laboratory before culture. A specimen was considered adequate when it contained >25 leukocytes and <10 epithelial cells per low-power field (LPF). Samples meeting these quality criteria were inoculated onto blood agar and MacConkey agar plates and incubated at 35–37 °C in an atmosphere containing 5% CO₂. A culture was classified as positive when at least one bacterial pathogen was recovered and reported by the clinical microbiology laboratory. Cultures from adequate sputum specimens in which no bacterial pathogen was recovered were classified as negative. The bacterial species recovered from positive cultures were recorded for analysis.

For the primary phenotype classification, the eosinophil percentage measured in the blood sample collected before systemic corticosteroid administration was dichotomized using a threshold of 2%. Patients with EOS% ≥ 2% were classified into the EOS group, whereas those with EOS% < 2 were classified into the non-EOS group. The 2% threshold was selected on the basis of its established use in previous studies of eosinophilic ECOPD and to facilitate comparison with published cohorts (9, 10). To assess the sensitivity of the findings to the eosinophil definition, we repeated the analyses of in-hospital mortality and ICU admission after reclassifying patients using EOS% thresholds of 3 and 4% and absolute blood eosinophil-count thresholds of 150 cells/μL (0.15 × 10^9^/L) and 300 cells/μL (0.30 × 10^9^/L). These alternative percentage- and absolute-count-based thresholds were selected on the basis of their established use in previous COPD studies and clinical trials (2, 11, 12). In our study, the primary outcome was in-hospital mortality, while key secondary outcomes included ICU admission, use of invasive mechanical ventilation during the hospitalization, use of non-invasive ventilation during the hospitalization, and length of hospital stay. ICU admission and the use of invasive or non-invasive ventilation were defined either as conditions present at the time of hospital admission or as events occurring during hospitalization.

This study was approved by the Medical Science Research Ethics Committee of our hospital (Approval No. M20250063). The requirement for informed consent was waived because of the retrospective study design. All included patients were analyzed anonymously, and all data were anonymized before analysis.

### 2.2 Statistical analysis

Data analysis was performed using SPSS 27.0 statistical software. The distribution of each continuous variable was assessed using the Shapiro–Wilk test. For continuous variables, if they followed a normal distribution, they were presented as mean ± standard deviation, and comparisons between two groups were made using an independent samples t-test. If the data did not follow a normal distribution, they were presented as median (interquartile range) [M (P25, P75)], and the Mann–Whitney U test was used for comparisons. Categorical variables were expressed as frequency (percentage) [*n* (%)], and comparisons between groups were performed using the χ^2^ test or Fisher’s exact test. Binary logistic regression was used to evaluate the associations of eosinophil status with in-hospital mortality and ICU admission. Two multivariable models were constructed for each outcome using prespecified sets of covariates with *P* valve <0.1 in the univariate analysis. Multicollinearity among the covariates included in each logistic regression model was assessed using variance inflation factors (VIFs) and tolerance values. Model calibration was visually evaluated using calibration plots comparing observed event proportions with mean predicted probabilities across predicted-risk groups. A generalized linear model was used to evaluate the association between eosinophil percentage and length of hospital stay. To examine whether the associations between eosinophil percentage and clinical outcomes were consistent across subsequent systemic corticosteroid treatment strata, we performed an exploratory stratified analysis based on whether patients received systemic corticosteroids during the hospitalization after collection of the baseline blood sample. Within each treatment stratum, clinical outcomes were compared between the EOS group (EOS% ≥ 2%) and the non-EOS group (EOS% < 2). To assess whether the findings were sensitive to the definition of the eosinophilic phenotype, we conducted additional threshold-based sensitivity analyses using EOS% thresholds of 3 and 4% and eosinophil-count thresholds of 150 cells/μL (0.15 × 10^9^/L) and 300 cells/μL (0.30 × 10^9^/L). Differences in in-hospital mortality and ICU admission between the threshold-defined groups were assessed using Pearson’s chi-square test. All statistical tests were two-sided, and a *p* value <0.05 was considered statistically significant.

## 3 Results

### 3.1 Baseline clinical characteristics

**Figure 1:**
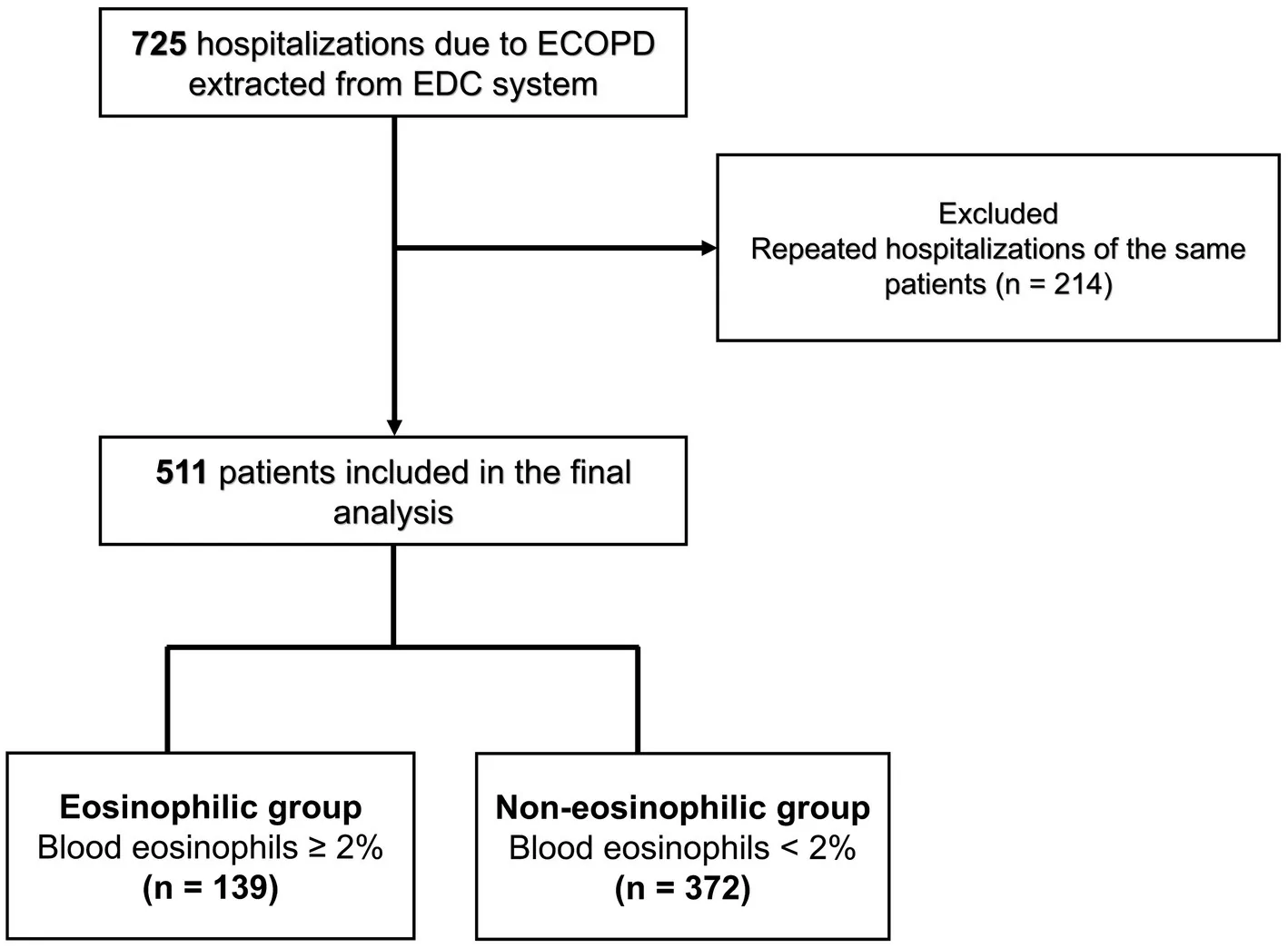
Flow diagram of patient selection and classification into the EOS and non-EOS groups.

The participant selection process was shown in Figure 1. A total of 511 patients were included in the final analysis, including 139 patients in the EOS group (27.2%) and 372 patients in the non-EOS group (72.8%). Baseline characteristics are summarized in Table 1. There were no statistically significant differences in gender and age between the two groups. Compared to the EOS group, the non-EOS group had significantly higher total peripheral white blood cell count, neutrophil count, and percentage, as well as lower lymphocyte count and percentage (*p* < 0.001). In addition, the non-EOS group had higher levels of CRP, NT-proBNP, procalcitonin, and D-dimer, as well as a higher incidence of heart failure, compared to the EOS group (*p* < 0.05). The spirometry function was comparable between two groups. Regarding medication treatment, there was no significant difference in the proportion of antibiotic use between the two groups. However, a higher proportion of patients in the non-EOS group received systemic corticosteroids (*p* < 0.001).

**Table 1:** Comparison of baseline characteristics between the two groups.

Variables	Total (*n* = 511)	EOS group (*n* = 139)	Non-EOS group (*n* = 372)	*P*
Age, (years)	78.00 (69.00, 84.00)	77.00 (69.00, 82.00)	78.00 (70.00, 84.00)	0.202
Gender, *n* (%)				0.434
Male	419 (82.00)	117 (84.17)	302 (81.18)	
Female	92 (18.00)	22 (15.83)	70 (18.82)	
Smoking status, *n* (%)				0.306
Current smoker	121 (23.68)	28 (20.14)	93 (25.00)	
Former smoker	318 (62.23)	94 (67.63)	224 (60.22)	
Never smoker	72 (14.09)	17 (12.23)	55 (14.78)	
FVC, (L)	2.08 (1.74, 2.62)	2.12 (1.85, 2.64)	2.07 (1.72, 2.59)	0.385
FEV_1_, (L)	1.04 (0.78, 1.40)	1.08 (0.84, 1.46)	1.03 (0.75, 1.35)	0.299
FVC%pred, (%)	64.70(16.08)	66.94(126.741)	41.40(15.71)	0.153
FEV_1_%pred, (%)	41.90(31.00, 54.3)	42.00(33.00,56.05)	41.40(30.00,53.00)	0.305
FEV_1_/FVC, (%)	50.00 (42.56, 59.00)	50.79 (43.69, 60.70)	50.00 (42.10, 58.00)	0.459
White blood cell count, (10^9/L)	7.76 (6.00, 10.41)	6.78 (5.46, 8.33)	8.32 (6.22, 11.67)	<0.001
Red blood cell count, (10^12/L)	4.29 (3.87, 4.75)	4.40 (3.84, 4.72)	4.27 (3.88, 4.75)	0.616
Hematocrit, (L/L)	0.40 (0.36, 0.44)	0.40 (0.37, 0.44)	0.40 (0.36, 0.44)	0.738
Hemoglobin, (g/L)	131.00 (118.00, 144.00)	133.00 (118.50, 143.50)	131.00 (117.75, 145.00)	0.891
Platelets, (10^9/L)	192.00 (153.00, 251.00)	200.00 (156.00, 250.50)	190.00 (153.00, 251.25)	0.309
Neutrophil count, (10^9/L)	6.17 (4.36, 8.57)	4.69 (3.59, 6.04)	6.85 (4.79, 9.69)	<0.001
Neutrophil percentage, (%)	0.79 (0.71, 0.86)	0.70 (0.62, 0.75)	0.83 (0.76, 0.88)	<0.001
Eosinophil count, (10^9/L)	0.05 (0.00, 0.15)	0.24 (0.17, 0.36)	0.02 (0.00, 0.07)	< 0.001
Eosinophil percentage	0.60% (0.00, 2.10%)	3.30% (2.50, 5.45%)	0.20% (0.00, 0.80%)	< 0.001
Lymphocyte count, (10^9/L)	0.96 (0.63, 1.40)	1.19 (0.88, 1.64)	0.86 (0.57, 1.28)	<0.001
Lymphocyte percentage, (%)	0.13 (0.08, 0.19)	0.18 (0.13, 0.25)	0.11 (0.07, 0.16)	<0.001
C-reactive protein, (mg/L)	1.88 (0.56, 8.38)	1.24 (0.37, 4.87)	2.23 (0.70, 9.96)	0.011
Procalcitonin, (ng/mL)	0.10 (0.05, 0.18)	0.07 (0.04, 0.11)	0.10 (0.05, 0.20)	<0.001
D-Dimer, (ug/mL)	0.29 (0.18, 0.61)	0.25 (0.16, 0.52)	0.32 (0.18, 0.66)	0.026
NT-proBNP, (pg/mL)	482.00 (157.50, 1690.50)	242.00 (85.50, 682.50)	561.50 (218.50, 2120.25)	<0.001
Hypertension, *n* (%)				0.915
No	237 (46.38)	65 (46.76)	172 (46.24)	
Yes	274 (53.62)	74 (53.24)	200 (53.76)	
Diabetes, *n* (%)				0.875
No	402 (78.67)	110 (79.14)	292 (78.49)	
Yes	109 (21.33)	29 (20.86)	80 (21.51)	
Heart failure, *n* (%)				0.006
No	448 (87.67)	131 (94.24)	317 (85.22)	
Yes	63 (12.33)	8 (5.76)	55 (14.78)	
Coronary heart disease, *n* (%)				0.967
No	405 (79.26)	110 (79.14)	295 (79.30)	
Yes	106 (20.74)	29 (20.86)	77 (20.70)	
Treatments during hospitalization				
Antibiotic use, *n* (%)				0.730
No	7 (1.37)	1 (0.72)	6 (1.61)	
Yes	504 (98.63)	138 (99.28)	366 (98.39)	
Systemic steroids, *n* (%)				0.001
No	263 (51.47)	88 (63.31)	175 (47.04)	
Yes	248 (48.53)	51 (36.69)	197 (52.96)	

Data are presented as median (25th percentile, 75th percentile) or *n* (%). FVC, forced vital capacity; FEV₁, forced expiratory volume in 1 s; % predicted, percentage of the predicted value.

### 3.2 Clinical outcomes

As shown in Table 2, the in-hospital mortality in the EOS group was significantly lower than that in the non-EOS group (1.44% vs. 6.72%, *p* = 0.018). The proportion of patients in the EOS group requiring ICU admission and the need for invasive or non-invasive ventilation were significantly lower than those in the non-EOS group (25.18% vs. 50.27%, *p* < 0.001; 2.88% vs. 7.80%, *p* = 0.044; 17.27% vs. 36.29%, *p* < 0.001). Additionally, the median length of hospital stay in the EOS group was shorter than that in the non-EOS group, but not statistically significant (11.0 days vs. 12.0 days, *p* = 0.057). Among the patients who receiving mechanical ventilation or non-invasive ventilation, there was no significant difference in the duration of mechanical ventilation or non-invasive ventilation between groups.

**Table 2:** Comparison of outcome indicators between the two groups.

Variables	Total (*n* = 511)	EOS group (*n* = 139)	Non-EOS group (n = 372)	*P*
Death, *n* (%)				0.018
No	484 (94.72)	137 (98.56)	347 (93.28)	
Yes	27 (5.28)	2 (1.44)	25 (6.72)	
ICU admission, *n* (%)				<0.001
No	289 (56.56)	104 (74.82)	185 (49.73)	
Yes	222 (43.44)	35 (25.18)	187 (50.27)	
Invasive mechanical ventilation, *n* (%)				0.044
No	478 (93.54)	135 (97.12)	343 (92.20)	
Yes	33 (6.46)	4 (2.88)	29 (7.80)	
Non-invasive mechanical ventilation, *n* (%)				<0.001
No	352 (68.88)	115 (82.73)	237 (63.71)	
Yes	159 (31.12)	24 (17.27)	135 (36.29)	
In-hospital invasive MV duration, h^a^	259.50 (112.50, 372.50)	297.50 (253.25, 566.25)	209.50 (100.00, 372.50)	0.363
In-hospital non-invasive MV duration, h^b^	124.50 (76.50, 211.00)	133.50 (39.25, 207.25)	124.00 (78.00, 212.50)	0.621
Length of hospital stay	12.00 (9.00, 15.00)	11.00 (8.00, 14.00)	12.00 (9.00, 16.00)	0.057

Data are presented median (25th percentile, 75th percentile) or n (%). ICU, intensive care unit; MV, mechanical ventilation; h, hours.

a 4 patients in the EOS group and 29 patients in the non-EOS group received mechanical ventilation. Duration of mechanical ventilation was compared in these patients between two groups.

b 24 patients in the EOS group and 135 patients in the non-EOS group received non-invasive ventilation. Duration of non-invasive ventilation was compared in these patients between two groups.

### 3.3 Microbial culture results between the two groups

As shown in Table 3, the detection rates of gram-positive bacilli and gram-negative bacilli were 9.35 and 15.11% in the EOS group, compared with 10.22 and 21.24% in the non-EOS group, respectively. The remaining patients (70.45%) had negative cultures from sputum specimens. There was no statistically significant difference in the total detection rate of Gram-positive or negative bacilli between the two groups. However, in terms of individual bacterial species, the detection rate of *Acinetobacter baumannii* was higher in the non-EOS group than in the EOS group (6.99% vs. 2.16%, *p* = 0.036).

**Table 3:** Comparison of microbiological results.

Variables (positive)	Total (*n* = 511)	EOS group (*n* = 139)	Non-EOS group (*n* = 372)	*P*
Gram-positive bacteria, n(%)	51 (9.98)	13 (9.35)	38 (10.22)	0.772
Coagulase-negative staphylococci, n(%)	22 (4.31)	4 (2.88)	18 (4.84)	0.331
*Streptococcus pneumoniae*, n(%)	15 (2.93)	6 (4.32)	9 (2.42)	0.403
*Enterococcus faecalis*, n(%)	4 (0.78)	2 (1.44)	2 (0.54)	0.642
*Staphylococcus aureus*, n(%)	9 (1.76)	1 (0.72)	8 (2.15)	0.474
Viridans streptococci, n(%)	1 (0.20)	0 (0.00)	1 (0.27)	1.000
*Streptococcus pyogenes*, n(%)	1 (0.20)	1 (0.72)	0 (0.00)	0.272
Gram-negative bacteria, n(%)	100 (19.57)	21 (15.11)	79 (21.24)	0.120
Klebsiella species, n(%)	23 (4.50)	5 (3.60)	18 (4.84)	0.547
*Pseudomonas aeruginosa*, n(%)	27 (5.28)	6 (4.32)	21(5.65)	0.550
*Stenotrophomonas maltophilia*, n(%)	13 (2.54)	4 (2.88)	9(2.42)	0.757
*Acinetobacter baumannii*, n(%)	29 (5.68)	3 (2.16)	26(6.99)	0.036
*Escherichia coli*, n(%)	5 (0.98)	2 (1.44)	3(0.81)	0.888
Unidentified Gram-negative bacilli, n(%)	21 (4.11)	8 (5.76)	13(3.50)	0.252
*Nocardia farcinica*, n(%)	1 (0.20)	0 (0.0)	1(0.27)	1.000

### 3.4 Multivariate analysis of clinical outcomes

#### 3.4.1 Mortality

As shown in Table 4, the univariable analysis showed that EOS ≥ 2% was associated with lower odds of in-hospital mortality (OR = 0.203, 95% CI, 0.047–0.867; *p* = 0.031). The other factors associated with mortality included age, ICU admission, white blood cell count, NT-proBNP levels, and heart failure (all *p* < 0.05). In multivariable Model 1, after adjustment for age and heart failure, EOS ≥ 2% remained associated with lower odds of in-hospital mortality (adjusted OR = 0.225, 95% CI, 0.052–0.971; *p* = 0.046). In Model 2, after adjustment for age, white blood cell count, and NT-proBNP, the association between EOS ≥ 2% and mortality was attenuated and was no longer statistically significant. The calibration plots showed generally close agreement between observed and predicted mortality within the observed range (Supplementary Figure S1).

**Table 4:** Analysis of risk factors affecting mortality in patients with ECOPD.

	Univariable		Multivariable-Model 1		Multivariable-Model 2	
	OR (95%CI)	*P*	OR (95%CI)	*P*	OR (95%CI)	*P*
Age	1.057 (1.010, 1.107)	0.017	1.050 (1.002, 1.101)	0.042	1.045 (0.994, 1.099)	0.085
Gender	0.964 (0.355, 2.617)	0.943				
EOS ≥ 2%	0.203 (0.047, 0.867)	0.031	0.225 (0.052, 0.971)	0.046	0.336 (0.071, 1.584)	0.168
White blood cell count	1.182 (1.094, 1.276)	<0.001			1.125 (1.034, 1.225)	0.006
Procalcitonin	1.008 (0.984, 1.033)	0.511				
NT-proBNP	1.000 (1.000, 1.000)	<0.001			1.000 (1.000, 1.000)	<0.001
Hypertension	0.793 (0.365, 1.723)	0.559				
Diabetes	1.311 (0.539, 3.186)	0.550				
Heart failure	2.675 (1.083, 6.610)	0.033	1.82 (0.725, 4.682)	0.199		
Coronary heart disease	1.361 (0.560, 3.310)	0.497				

#### 3.4.2 ICU admission

For ICU admission, univariate logistic regression analysis showed that age, gender, EOS ≥ 2%, white blood cell count, procalcitonin (PCT), and NT-proBNP were associated with ICU admission. After adjusting for age, gender, heart failure in the multivariate logistic regression model 1, EOS ≥ 2% remained associated with lower odds of ICU admission (adjusted OR = 0.357, 95% CI, 0.229–0.556; *p* < 0.001). Additionally, age and heart failure were associated with an increased risk of ICU admission. In Model 2, after adjustment for age, white blood cell count, PCT, and NT-proBNP, EOS ≥ 2% remained associated with lower odds of ICU admission (adjusted OR = 0.377, 95% CI, 0.231–0.613; *p* < 0.001) (Table 5). Across the four logistic regression models, variance inflation factors (VIFs) ranged from 1.005 to 1.083, while tolerance values ranged from 0.923 to 0.995, indicating no evidence of problematic multicollinearity among the covariates included in the mortality or ICU admission models (Supplementary Table S1). The calibration plots demonstrated acceptable agreement between the predicted probabilities and observed ICU admission rates across the observed prediction range, although some variability was evident in both models. (Supplementary Figure S2).

**Table 5:** Analysis of risk factors affecting the secondary outcome—ICU Admission in patients with ECOPD.

	Univariable		Multivariable-Model 1		Multivariable-Model 2	
	OR (95%CI)	*P*	OR (95%CI)	*P*	OR (95%CI)	*P*
Age	1.038 (1.019, 1.058)	<0.001	1.029 (1.009, 1.050)	0.005	1.025 (1.004, 1.048)	0.022
Gender	0.525 (0.333, 0.829)	0.006	0.692 (0.421, 1.136)	0.145		
EOS ≥ 2%	0.333 (0.216, 0.514)	<0.001	0.357 (0.229, 0.556)	<0.001	0.377 (0.231, 0.613)	<0.001
White blood cell count	1.092 (1.041, 1.145)	<0.001			1.057 (1.001, 1.117)	0.044
Procalcitonin	1.495 (1.129, 1.981)	0.005			1.294 (1.053, 1.590)	0.014
NT-proBNP	1.000 (1.000, 1.000)	<0.001			1.000 (1.000, 1.000)	<0.001
Hypertension	1.211 (0.852, 1.721)	0.286				
Diabetes	1.133 (0.741, 1.734)	0.564				
Heart failure	3.231 (1.840, 5.674)	<0.001	2.313 (1.282, 4.173)	0.005		
Coronary heart disease	0.998 (0.648, 1.536)	0.991				

#### 3.4.3 Length of hospital stay

Generalized linear model analysis revealed that the EOS group had a shorter hospital stay (IRR = 0.879, 95% CI: 0.794–0.973, *p* = 0.013). In multivariate Model 1, after adjustment for age and heart failure, EOS ≥ 2% remained associated with a shorter length of stay (adjusted IRR = 0.898, 95% CI, 0.810–0.995; *p* = 0.041). However, in Model 2, after adjustment for age, white blood cell count, and NT-proBNP, the association between EOS ≥ 2% and length of stay was attenuated and was no longer statistically significant (adjusted IRR = 0.910, 95% CI, 0.817–1.013; *p* = 0.083) (Table 6).

**Table 6:** Analysis of risk factors affecting length of hospital stay in patients with ECOPD.

	Univariable		Multivariable-Model 1		Multivariable-Model 2	
	IRR (95%CI)	*P*	IRR (95%CI)	*P*	IRR (95%CI)	*P*
Age	1.009 (1.004, 1.013)	<0.001	1.007 (1.002, 1.012)	0.005	1.008 (1.003, 1.013)	0.001
Gender	0.989 (0.878, 1.113)	0.851				
EOS ≥ 2%	0.879 (0.794, 0.973)	0.013	0.898 (0.810, 0.995)	0.041	0.910 (0.817, 1.013)	0.083
White blood cell count	1.018 (1.006, 1.031)	0.004			1.011 (0.999, 1.023)	0.083
Procalcitonin	0.999 (0.993, 1.004)	0.581				
NT-proBNP	1.000 (1.000, 1.000)	<0.001			1.000 (1.000, 1.000)	<0.001
Hypertension	1.090 (0.995, 1.194)	0.064				
Diabetes	0.926 (0.829, 1.035)	0.177				
Heart failure	1.295 (1.129, 1.485)	<0.001	1.240 (1.081, 1.422)	0.002		
Coronary heart disease	1.023 (0.914, 1.144)	0.697				

#### 3.4.4 Clinical outcomes stratified by systemic corticosteroid treatment

Among the 263 patients who did not receive systemic corticosteroids, the EOS group had lower rates of in-hospital mortality (1.14% vs. 8.00%, *p* = 0.024), ICU admission (27.27% vs. 52.00%, *p* < 0.001), and non-invasive mechanical ventilation (20.45% vs. 35.43%, *p* = 0.013) than the non-EOS group. The EOS group also had a shorter median length of hospital stay (10.5 [8.0–14.0] vs. 13.0 [9.0–18.0] days, *p* = 0.008). The rate of invasive mechanical ventilation was numerically lower in the EOS group, although the difference did not reach statistical significance (3.41% vs. 10.29%, *p* = 0.052).

Among the 248 patients who received systemic corticosteroids, the EOS group had lower rates of ICU admission (21.57% vs. 48.73%, *p* < 0.001) and non-invasive mechanical ventilation (11.76% vs. 37.06%, *p* < 0.001). No statistically significant differences were observed in in-hospital mortality, invasive mechanical ventilation, or length of hospital stay. Overall, within-stratum comparisons showed lower ICU admission and non-invasive mechanical ventilation rates in the EOS group in both systemic corticosteroid treatment strata, whereas differences in mortality and length of hospital stay reached statistical significance only among patients who did not receive systemic corticosteroids.

In addition to the within-stratum comparisons, we formally evaluated the interaction between EOS status and subsequent systemic corticosteroid treatment for each clinical outcome. No statistically significant interactions were observed for in-hospital mortality (*P* for interaction = 0.527), ICU admission (P for interaction = 0.700), invasive mechanical ventilation (*P* for interaction = 0.939), non-invasive ventilation (P for interaction = 0.188), or length of hospital stay (*P* for interaction = 0.222). Thus, the associations between EOS and clinical outcomes were not influenced by systemic corticosteroid treatment strata (Supplementary Table S2).

#### 3.4.5 Sensitivity analysis

In the threshold-based analyses, the EOS-high group defined by the primary EOS% threshold of 2% had a lower in-hospital mortality rate than the EOS-low group (*p* = 0.018). No statistically significant differences in mortality were observed at the alternative thresholds of EOS% ≥ 3%, EOS% ≥ 4%, an absolute eosinophil count ≥150 cells/μL, or an absolute eosinophil count ≥300 cells/μL (all *p* > 0.05; Supplementary Table S3). For ICU admission, the EOS-high group had lower admission rates at the thresholds of EOS% ≥ 2% (25.2% vs. 50.3%, *p* < 0.001), EOS% ≥ 3% (31.0% vs. 45.9%, *p* = 0.012), EOS% ≥ 4% (29.1% vs. 45.2%, *p* = 0.023), and an EOS count ≥150 cells/μL (31.3% vs. 47.5%, *p* = 0.001). At the EOS count threshold of 300 cells/μL, the ICU admission rate was lower in the EOS-high group, but the difference did not reach statistical significance (30.8% vs. 44.9%, *p* = 0.052) (Supplementary Table S4).

## 4 Discussion

This study found that, compared to patients with EOS < 2%, those with an EOS ≥ 2% in peripheral blood during ECOPD had a lower risk of ICU admission, and this association was independent. This suggested that patients with higher EOS levels may have better prognosis or therapeutic response after admission, thereby reducing the likelihood of disease progression. These results were consistent with our previous pilot study, which showed that ECOPD patients with increased eosinophils had better clinical outcomes during hospitalization, including shorter hospital stays, lower incidence of respiratory failure, and reduced ICU admission rates (13). A retrospective study involving 217 ECOPD patients also indicated a significant reduction in ICU admission rates in the EOS group (14). Similarly, a retrospective analysis of 2,406 ECOPD patients showed that, after multivariate adjustment, the ICU admission risk in the EOS group was significantly lower than that in the non-EOS group (OR = 0.34, 95% CI: 0.14–0.82) (15). Additionally, a cross-sectional study categorized patients into EOS (>5%) and non-EOS (≤5%) groups and found that the ICU admission rate in the EOS eosinophil group was significantly lower (9.4% vs. 21.7%, *p* = 0.018). The non-EOS group had higher mechanical ventilation requirements and longer hospital stays, further suggesting that low EOS levels may be associated with disease severity (16). However, there were some inconsistent results in other studies. For example, a prospective study involving 973 patients did not observe a significant difference in ICU admission rates based on eosinophil levels (17), suggesting that this association might be influenced by study population, treatment protocols, or other confounding factors, and warranted further exploration in future research. Low eosinophil levels were often associated with a neutrophil-dominant inflammatory response, which was typically linked to more significant bacterial infections and airway tissue damage, leading to higher risks of respiratory failure and ICU admission (18, 19). Several studies further suggested that ECOPD patients with elevated eosinophil levels often exhibited a decrease in systemic inflammation markers, which were consistent with our study. Research had shown that in ECOPD patients with increased eosinophils, levels of D-dimer, C-reactive protein (CRP), and procalcitonin (PCT) were significantly lower (20). Two other studies also indicated significantly lower CRP and neutrophil counts in these patients (15, 21). A large multi-center study also confirmed that the EOS group patients had significantly lower levels of CRP, fibrinogen, D-dimer, and PCT (*p* < 0.001) (18). These findings suggested that low EOS levels often reflect a stronger systemic inflammation, which was closely related to higher mechanical ventilation demands and ICU admission rates (22). Our study further supported the association between increased EOS and lower ICU admission rates in ECOPD patients, providing more insights for using peripheral blood EOS level as a biomarker for assessing ECOPD prognosis. These findings contributed to a better understanding of the clinical significance and potential mechanisms of EOS during ECOPD.

This study also found that EOS% ≥ 2% was associated with lower in-hospital mortality in patients with ECOPD. In the model adjusted for age and heart failure, EOS% ≥ 2% remained independently associated with lower odds of in-hospital mortality. However, their association did not remain significant in the sensitivity analysis. Some previous studies demonstrated that high blood EOS counts (≥ 300 cells/μL) were associated with lower in-hospital mortality and reduced mechanical ventilation requirements in ECOPD patients, indicating significantly better short-term outcomes compared to the non-EOS group (23, 24). However, a large-scale prospective cohort study in China (*n* = 12,831) found that there were no statistically significant differences in mortality rates between the two groups within the ICU subgroup (18). Furthermore, a systematic review indicated that high EOS levels in COPD patients were positively correlated with increased frequency of exacerbations and mortality, despite heterogeneity across different populations and studies (25). Therefore, there was still some inconsistency in the related evidence. Differences in the results of various studies may stem from discrepancies in inclusion criteria, disease severity distribution, treatment regimens (especially corticosteroid use), and the timing and threshold definitions of EOS measurement. The effect of EOS on COPD may differ between the stable and exacerbation stages, where increased EOS levels may increase the risk of exacerbation during the stable stage, while during the exacerbation stage, EOS tends to serve as a marker predicting better clinical outcomes. The mechanisms behind this need to be explored through further in-depth studies.

In this study, there was no significant statistical difference in the overall detection rates of Gram-positive and Gram-negative bacteria between the two groups. However, further analysis of individual bacterial species revealed that the detection rate of *Acinetobacter baumannii* was significantly higher in the non-EOS group compared to the EOS group. Existing studies generally suggested that low EOS levels were associated with higher bacterial infection rates. For instance, Choi et al. found that in ECOPD patients, the bacterial pathogen detection rate was significantly higher in the non-EOS group compared to the EOS group (OR = 1.744, 95% CI 1.107–2.749, *p* = 0.017) (26). Additionally, in a study involving 596 ECOPD patients, the non-EOS group had significantly higher levels of Gram-positive bacteria in sputum cultures, particularly Staphylococcus (*p* = 0.006) and Enterococcus (*p* = 0.013) (27). Beyond culture-based findings, accumulating microbiome studies indicated that eosinophilic ECOPD represented a distinct airway microbial endotype compared with the “bacterial infection/neutrophilic” endotype. Specifically, eosinophilic exacerbations tended to be enriched in Firmicutes (with Streptococcus often predominating) and showed a lower relative abundance of *Haemophilus influenzae*, whereas the bacterial infection phenotype was more frequently dominated by Proteobacteria, particularly *H. influenzae* and *Moraxella catarrhalis*. Moreover, a reduced Proteobacteria-to-Firmicutes ratio had been described in eosinophilic phenotypes during exacerbations (28). Similar results had also been observed in stable COPD, where eosinophilic phenotype (e.g., blood eosinophils >100 cells/μL) was more commonly characterized by Firmicutes predominance, while non-eosinophilic phenotype was more often Proteobacteria-predominant with increased *H. influenzae* (29). A recent multicohort longitudinal analysis further suggested that eosinophilic patients exhibit lower proportions of *H. influenzae* and *M. catarrhalis* than neutrophilic patients, with partial microbiome stability maintained across exacerbations in a subset of eosinophilic cases (6). Collectively, these findings supported the concept that EOS-related inflammatory endotypes are linked to distinct airway microbiota profiles. Future research should conduct more detailed analyses of Gram-negative bacteria, bacterial strain characteristics, antibiotic use, and factors related to hospital/ICU environments to further clarify the potential impact of EOS levels on specific bacterial infections, thereby providing more reliable clinical evidence.

The threshold value of the blood EOS holds significant clinical value, directly impacting phenotype classification and guiding the use of inhaled glucocorticoids. There is still variability in the threshold settings for EOS percentage in the existing literature, with common cut-off values including 2, 3, and 4%, especially in patients with ECOPD. Among these, several studies had chosen 2% as the threshold for blood EOS. A multi-center retrospective study found that an EOS ≥ 2% in the blood of patients with ECOPD served as the optimal cut-off value for predicting eosinophilic exacerbation, demonstrating good specificity (around 80%), though its sensitivity was relatively low (ranging from 39 to 45%) (30). Additionally, another systematic review confirmed that a blood EOS ≥ 2% or ≥300 cells/μL could significantly predict the risk of moderate to severe exacerbations in COPD (31). However, some studies also used 3 and 4% as thresholds, but their predictive value was relatively weaker. In a study, the 3% threshold did not show significant predictive effects, while the 4% threshold slightly enhanced risk prediction, although its statistical significance was limited due to a small sample size (31). Therefore, the majority of current literature tended to use EOS ≥ 2% as the standard threshold, which was also used in our study. Moreover, the stability of blood EOS levels is also an important issue to consider. A retrospective study from Belgium pointed out that EOS counts in individual patients could vary over time, and a single measurement may have limitations. This variability reduced the reliability of using a single EOS percentage as a predictive tool (32). Overall, the EOS threshold is crucial for phenotype classification and clinical outcome assessment. So far, using 2% or 300 cells/μL as the cut-off for blood eosinophils helps to effectively identify patients with eosinophilic exacerbations, while the determination of the cut-off value and the dynamic analysis of EOS level still need further research.

This study had several limitations: (1) The single-center retrospective design may have introduced selection bias and limits the generalizability of the findings. (2) Because only in-hospital outcomes were assessed, the present results cannot be extrapolated to long-term mortality, readmission or subsequent exacerbations. Moreover, the observed associations were not externally validated. Prospective multicenter studies with longitudinal follow-up and independent external validation are therefore warranted. (3) This study used traditional lower respiratory tract bacterial culture methods to assess the pathogen spectrum. However, traditional culture techniques have limited sensitivity for detecting certain difficult-to-culture microorganisms, viruses, and fungi, and may not fully reflect the overall microbial ecosystem of the lower respiratory tract. (4) The patients included in this study were all hospitalized ECOPD patients prior to 2023, and did not include patients who have been treated with biologic therapies (such as dupilumab and mepolizumab) that have been recently used in clinical practice. Therefore, the study results may not fully reflect the characteristics of ECOPD in the context of modern treatments. Future research will focus more on this patient population. (5) Although blood samples were collected before systemic corticosteroid administration, the potential influence of pre-admission maintenance ICS therapy cannot be completely excluded. Previous evidence suggests that ICS has only a small average effect on blood eosinophil counts in patients with COPD (33), nevertheless, residual confounding related to differences in ICS dose, duration, adherence, and individual response may remain. (6) In addition, antibiotic exposure before hospital admission may have reduced bacterial culture yield. Because the type, duration, and timing of antibiotic use before hospital admission were not reliably documented, the extent of this potential bias could not be quantified.

In conclusion, this study showed that ECOPD patients with EOS ≥ 2% had more favorable in-hospital clinical profiles, including lower rates of ICU admission, mechanical ventilation and mortality, shorter hospital stays, and lower levels of inflammatory markers.

## Data Availability

The raw data supporting the conclusions of this article will be made available by the authors, without undue reservation.
