# Supplementary material for: Systemic inflammation, microbiology, and clinical outcomes in eosinophilic and non-eosinophilic acute exacerbations of chronic obstructive pulmonary disease
Source: Front Med (Lausanne). 2026 Aug 27;13:1917271. doi: 10.3389/fmed.2026.1917271 (PMC13558021; doi:10.3389/fmed.2026.1917271)
Supplement: Supplementary file 1 [file Supplementary_file_1.docx]

**Supplemental materials**

**Title: Systemic Inflammation, Microbiology, and Clinical Outcomes in Eosinophilic and Non-Eosinophilic Acute Exacerbations of Chronic Obstructive Pulmonary Disease**

Catalog

[Supplementary Figure S1. Calibration Curves for Mortality Models	3](#_Toc236491703)

[Supplementary Figure S2. Calibration Curves for ICU-Admission Models	4](#_Toc236491704)

[Supplementary Table S1. Collinearity Diagnostics for Logistic Regression Models	5](#_Toc236491705)

[Supplementary Table S2. Clinical Outcomes According to Eosinophil Status, Stratified by In-Hospital Systemic Corticosteroid Treatment	6](#_Toc236491706)

[Supplementary Table S3. Associations of Eosinophil Thresholds with Mortality	7](#_Toc236491707)

[Supplementary Table S4 Associations of Eosinophil Thresholds with ICU Admission	7](#_Toc236491708)

# Supplementary Figure S1. Calibration Curves for Mortality Models

# Supplementary Figure S2. Calibration Curves for ICU-Admission Models

# Supplementary Table S1. Collinearity Diagnostics for Logistic Regression Models

| Model | Variable | VIF | Tolerance |
| --- | --- | --- | --- |
| Mortality Model 1 | Age | 1.038 | 0.963 |
| Mortality Model 1 | EOS ≥2% | 1.016 | 0.985 |
| Mortality Model 1 | Heart failure | 1.052 | 0.951 |
| Mortality Model 2 | Age | 1.020 | 0.981 |
| Mortality Model 2 | EOS ≥2% | 1.068 | 0.937 |
| Mortality Model 2 | White blood cell count | 1.083 | 0.924 |
| Mortality Model 2 | NT-proBNP | 1.041 | 0.961 |
| ICU Model 1 | Age | 1.083 | 0.923 |
| ICU Model 1 | Gender | 1.079 | 0.927 |
| ICU Model 1 | EOS ≥2% | 1.016 | 0.985 |
| ICU Model 1 | Heart failure | 1.073 | 0.932 |
| ICU Model 2 | Age | 1.019 | 0.981 |
| ICU Model 2 | EOS ≥2% | 1.069 | 0.936 |
| ICU Model 2 | White blood cell count | 1.082 | 0.924 |
| ICU Model 2 | Procalcitonin | 1.005 | 0.995 |
| ICU Model 2 | NT-proBNP | 1.041 | 0.960 |

# Supplementary Table S2. Clinical Outcomes According to Eosinophil Status, Stratified by In-Hospital Systemic Corticosteroid Treatment

| **Outcome** | **No systemic corticosteroid treatment (n=263)** | | ***P* value** | **Systemic corticosteroid treatment (n=248)** | | ***P* value** | ***P* for interaction** |
| --- | --- | --- | --- | --- | --- | --- | --- |
|  | **EOS group (n=88)** | **Non-EOS group (n=175)** |  | **EOS group (n=51)** | **Non-EOS group (n=197)** |  |  |
| **In-hospital mortality, n (%)** | 1 (1.14) | 14 (8.00) | 0.024 | 1 (1.96) | 11 (5.58) | 0.479 | 0.527 |
| **ICU admission, n (%)** | 24 (27.27) | 91 (52.00) | <0.001 | 11 (21.57) | 96 (48.73) | <0.001 | 0.700 |
| **In-hospital invasive mechanical ventilation, n (%)** | 3 (3.41) | 18 (10.29) | 0.052 | 1 (1.96) | 11 (5.58) | 0.479 | 0.939 |
| **In-hospital non-invasive ventilation, n (%)** | 18 (20.45) | 62 (35.43) | 0.013 | 6 (11.76) | 73 (37.06) | <0.001 | 0.188 |
| **Length of hospital stay, days** | 10.5 (8.0–14.0) | 13.0 (9.0–18.0) | 0.008 | 12.0 (9.0–15.5) | 12.0 (9.0–15.0) | 0.683 | 0.222 |

P values for interaction assess whether the associations between EOS status and clinical outcomes differ according to systemic corticosteroid treatment.

# Supplementary Table S3. Associations of Eosinophil Thresholds with Mortality

| Threshold | EOS-high, events/n | EOS-low, events/n | Pearson χ² | *P* |
| --- | --- | --- | --- | --- |
| EOS percentage ≥2% (primary) | 2/139 | 25/372 | 5.640 | 0.018 |
| EOS percentage ≥3% | 2/84 | 25/427 | 1.693 | 0.193 |
| EOS percentage ≥4% | 2/55 | 25/456 | 0.334 | 0.563 |
| EOS count ≥150 cells/μL | 3/128 | 24/383 | 2.950 | 0.086 |
| EOS count ≥300 cells/μL | 2/52 | 25/459 | 0.239 | 0.625 |

# Supplementary Table S4 Associations of Eosinophil Thresholds with ICU Admission

| Threshold | EOS-high, events/n | EOS-low, events/n | Pearson χ² | *P* |
| --- | --- | --- | --- | --- |
| EOS percentage ≥2% (primary) | 35/139 | 187/372 | 25.923 | <0.001 |
| EOS percentage ≥3% | 26/84 | 196/427 | 6.384 | 0.012 |
| EOS percentage ≥4% | 16/55 | 206/456 | 5.168 | 0.023 |
| EOS count ≥150 cells/μL | 40/128 | 182/383 | 10.336 | 0.001 |
| EOS count ≥300 cells/μL | 16/52 | 206/459 | 3.785 | 0.052 |
